# Effect of involvement of midwives in maternal care on patient and provider satisfaction in secondary-level public health facilities in Bangladesh: a comparative quasi-experimental study

**DOI:** 10.7189/jogh.15.04183

**Published:** 2025-07-04

**Authors:** Hassan Rushekh Mahmood, Lubna Hossain, Abu Sayeed, Farhia Azrin, Trisha Mallick, Tanvir Hayder, Anisuddin Ahmed, Sabrina Jabeen, Tajrin Tahrin Tonmon, Md Mahiur Rahman, Mahmudul Hassan AKM, Md Abu Bakkar Siddique, Shamsuz Zaman, Afruna Rahman, Haroon Bin Murshid, Nuzhat Nadia, Mustufa Mahmud, Md Azizul Alim, Dewan Md Emdadul Hoque, Abu Sayed Md Hasan, Shams El Arifeen, Ahmed Ehsanur Rahman, Vibhavendra S Rasghuvanshi

**Affiliations:** 1 Maternal and Child Health Division, International Centre for Diarrhoeal Disease Research, Bangladesh, Dhaka, Bangladesh; 2 Global Health and Migration Unit, Department of Women's and Children's Health, Uppsala University, Uppsala, Sweden; 3 United Nations Population Fund, Dhaka, Bangladesh; 4 Infectious Diseases Division, International Centre for Diarrhoeal Disease Research, Bangladesh, Dhaka, Bangladesh; 5 Directorate General of Health Services, Ministry of Health and Family Welfare, Maternal, Newborn, Child, and Adolescent Health, Dhaka, Bangladesh; 6 United Nations Children’s Fund, Dhaka, Bangladesh

## Abstract

**Background:**

Maternity care satisfaction for pregnant women and recently delivered mothers is linked to both short- and long-term complications. For health care providers, dissatisfaction can lead to high turnover, reduced efficiency, and decreased service quality. Increased midwifery care coverage enhances satisfaction; however, the Government of Bangladesh-sanctioned midwife positions are limited to the primary health care level only, not extending to secondary or tertiary health tiers. We aimed to evaluate the impact of introducing midwifery care at secondary-level health care facilities in Bangladesh by comparing the satisfaction levels of both providers and patients with those receiving regular care.

**Methods:**

We conducted a quasi-experimental study from September 2021 to December 2023 at two secondary-level health facilities – Sunamganj District Hospital (DH) (intervention) and Khagrachari DH (control). The primary outcome was the mean (x̄) percentage score of maternal satisfaction with antenatal care (ANC), delivery, and postnatal care (PNC) services, along with providers’ satisfaction regarding their working environment, training, logistical support, and overall patient and service satisfaction at both facilities.

**Results:**

We screened for eligibility a total of 200 pregnant women or recently delivered mothers who visited the facility for ANC, delivery, or PNC services. The satisfaction scores were significantly higher among women who received midwifery care compared to those who received regular care. The scores were for ANC x̄ = 97.8 (95% confidence interval (CI) = 96.6–99.0) *vs.* x̄ = 71.0 (95% CI = 68.7–73.4), for delivery x̄ = 97.9 (95% CI = 97.4–98.3) *vs.* x̄ = 83.3 (95% CI = 80.2–86.3), and for PNC x̄ = 97.5 (95% CI = 96.5–98.4) *vs.* x̄ = 77.7 (95% CI = 73.8–81.6). Similarly, the satisfaction score among midwives was significantly higher than among providers in regular care facilities (x̄ = 88.7; 95% CI = 82.9–94.5 *vs.* x̄ = 77.7; 95% CI = 73.0–82.3).

**Conclusions:**

Our study highlights that midwifery care significantly enhances patient and provider satisfaction compared to regular care across antenatal, delivery, and postnatal services.

Daily, 6500 newborn deaths and 810 maternal deaths occur worldwide, and more than 90% of these deaths occur in low- and middle-income countries (LMICs), mainly from complications related to pregnancy and childbirth [1–3]. In Bangladesh, almost 14 mothers are dying every day due to these complications [4]. One of the leading causes of these deaths is the lack of quality treatment during the antenatal, delivery and postnatal period of pregnant mothers [5–7]. Studies have shown that receiving care from skilled health workers before, during, and after childbirth can reduce the number of complications and mortality among mothers [8]. In high-income countries, women receive comprehensive care from skilled health care workers, including antenatal care (ANC), labour care, and postnatal care. This continuous and high-quality medical support significantly reduces maternal and newborn mortality rates [9]. On the other hand, a study in LMICs indicated that about 94% of preventable maternal deaths occur as a result of the inadequate availability of skilled health care workers in health facilities [10].

As a solution to the shortage of skilled health care workers in LMICs, the World Health Organization (WHO) recognises the critical role of midwives in providing quality care throughout pregnancy, birth, and the postnatal period to prevent maternal and newborn complications [7]. As per the International Confederation of Midwives (ICM), under midwifery care, a qualified midwife provides comprehensive care to pregnant women during all stages of maternal care, including the antenatal, delivery, and postnatal phases, to reduce maternal and newborn complications [11]. Additionally, midwifery-led care, a patient-centred care, has been associated with lower rates of preterm births, reduced the need for interventions, and improved women’s experience of care as well as clinical outcomes [12–14]. According to the 2014 Lancet Series on Midwifery, midwifery interventions can decrease mother and neonatal deaths in LMICs by 67% and 64%, respectively, depending on the extent of coverage, resulting in more than four million lives being saved every year by 2035 [15].

In 2013, the Government of Bangladesh initiated the three-year midwifery diploma course based on ICM standards, leading to the national deployment of licensed midwives only at the subdistrict level (primary level) in 2016 [16]. While increased midwifery care coverage can reduce maternal and neonatal death, limiting midwife deployment to the primary level remains a significant barrier to further reducing maternal and infant mortality in Bangladesh [15]. The quality of midwifery care is also affected by challenges such as an insufficient workforce, excessive workloads, hospital overcrowding, and a lack of logistical support, including necessary supplies and medications in Bangladesh [17–21]. These issues contribute to dissatisfaction among midwives, nurses working with midwives, pregnant women, and recently delivered mothers, which is the leading cause of high turnover rates, reduced workplace efficiency, and a decline in service quality [22]. To address these challenges, the WHO encourages the presence of qualified birth attendants at every delivery and recommends measuring women's satisfaction to enhance the quality and effectiveness of health services [23].

For pregnant women and recently delivered mothers, maternity care satisfaction is associated with various short- and long-term complications [24]. In 2015, Srivastava et al. reported that receiving kind and non-abusive treatment, regardless of one’s sociocultural or economic background, is particularly necessary for women's health [25]. Thus, the satisfaction of midwives, pregnant women and recently delivered mothers while receiving or providing maternal care is an important indicator of the quality of maternal care [24]. However, none of the currently available literature focuses on the satisfaction of both providers and recipients of maternal care in Bangladesh.

Therefore, we aimed to measure the effect of introducing midwifery care at secondary-level health care facilities by assessing the satisfaction of both providers and pregnant women, as well as recently delivered mothers, comparing midwifery care to regular care in these health care facilities in Bangladesh.

## METHODS

### Study design and setting

We conducted a quasi-experimental study design from September 2021 to December 2023 in two district hospitals (DH), which are secondary-level health facilities (Sunamganj DH and Khagrachari DH). We purposively selected the two facilities based on the recommendations from the Maternal, Newborn, Child, and Adolescent Health programme of the Directorate General of Health Services, Ministry of Health and Family Welfare, Bangladesh. These facilities were recommended by the Directorate General of Health Services due to their strategic location, serving as key health care hubs for hard-to-reach and underserved populations, making them ideal for implementing interventions to improve access to quality care.

### Study population

Our primary study population was pregnant women who received ANC, recently delivered mothers who gave birth at the study sites, and the same mothers who received their first postnatal care (PNC) before discharge during the study period. Our secondary population consisted of frontline service providers, including midwives and nurses (who worked directly with midwives in the ANC corner and delivery room) in both facilities during the study period.

### Eligibility criteria

All pregnant women at the ANC visit, recently delivered mothers and mothers receiving first postnatal care before discharge were eligible for the study. From the intervention site, we only enrolled patients who received services from the midwives.

We excluded from the study pregnant women or mothers with serious medical complications like hypertension and cardiac problems, diabetes mellitus, renal and respiratory disease, and thyroid disease and mothers who had caesarean delivery.

### Intervention and control facility

We chose the intervention and control facility by lottery between the selected two facilities. Sunamganj DH served as the intervention facility, and Khagrachari DH served as the control facility. In the intervention facility, the United Nations Population Fund deployed six midwives with the existing staff for the three corners mentioned. We organised the midwives in the intervention facility into shifts across the ANC/PNC corner and the delivery room. The midwives worked morning, evening, and night shifts, ensuring 24-hour coverage, while one midwife had a day off (Figure S1 in the **Online Supplementary Document**).

### Midwives’ job responsibilities in the intervention site

Midwives are crucial in providing comprehensive care to women in the ANC, delivery, and PNC corners of a health facility. Their responsibilities spanned from routine check-ups to emergency interventions, ensuring the well-being of both the mother and the newborn. Here is a detailed description of their duties (Figure S2 in the **Online Supplementary Document**) across three key areas, as presented in the following text.

### Antenatal care

During the antenatal period, study midwives focused on monitoring the health of the mother and foetus, preparing the mother for childbirth, and identifying any potential complications in the ANC corner.

Midwives conduct routine health assessments and monitor the mother’s health, which includes checking blood pressure, weight, and abdominal measurements. They also conduct physical examinations to identify high-risk pregnancies, such as pre-existing medical conditions (diabetes, high blood pressure, autoimmune diseases, or depression), pregnancy-related conditions (gestational diabetes, pre-eclampsia, anaemia, gestational diabetes, foetal growth) multiple pregnancies (carrying twins, triplets) previous pregnancy complications (including preterm labour, multiple miscarriages, or having a child with a genetic condition or birth defect). They provided health education and counselling on nutrition and lifestyle, including healthy eating, exercise, and lifestyle modifications that benefited both the mother and the foetus. Additionally, midwives offered emotional support and counselled according to the national maternal standard operating procedures on diet, the importance of good nutrition, adequate weight gain, iron, folic acid, tablet calcium, regular ANC visits, maternal danger signs, tetanus toxoid vaccine and the importance of exercise. They also advised on postpartum family planning, personal hygiene, essential newborn care, and immediate and exclusive breastfeeding benefits. In addition to these, they also performed preventive care and risk management of high-risk mothers, such as multiple pregnancies, pre-eclampsia, gestational diabetes mellitus, and so on. They referred the mother to specialised care when necessary.

### Delivery (intrapartum care)

Midwives managed the childbirth process, ensuring a safe and positive experience for the mother and baby during labour and delivery. They continuously monitored the mother’s vital signs, including blood pressure, pulse, and temperature, as well as uterine contractions, cervical dilation, and the foetal heart rate, to identify any signs of distress or complications for both mother and baby. In the event of emergencies, such as prolonged labour, foetal distress, or postpartum haemorrhage, midwives initiated emergency interventions, including administering oxytocin for uterine contraction, performing manual removal of the placenta, providing intravenous fluids, and coordinating with obstetricians for timely caesarean sections or other advanced care when necessary. They also assessed the newborn’s health (Apgar score), initiated skin-to-skin contact, assisted with the first breastfeeding immediately after delivery, and ensured prompt neonatal resuscitation if required.

### Postnatal care

In the postnatal period, midwives continued to provide essential care to the mother and newborn. They performed the mother’s health monitoring for physical recovery, including uterine involution, vaginal discharge (lochia) and bleeding, and the healing of any perineal wounds or surgical incisions (*e.g.* from a caesarean section) and provided advice on managing common complications. They also assisted with breastfeeding and screened for postnatal depression or anxiety and offered appropriate support or referrals to the mothers.

Additionally, midwives conducted newborn care and monitored the newborn’s weight, feeding patterns, and overall health screening for common neonatal complications, and counselled parents and family members on the importance of the expanded programme on immunisation schedule and referred them to the expanded programme on immunisation corners.

Additionally, they also advised the mother on health education and family planning, including information on contraception methods. They offered guidance on postpartum exercises, nutrition, and hygiene, as well as advice on sexual health and when it was safe to resume sexual activity. To monitor the ongoing health of both the mother and the baby, midwives conducted follow-up care visits and referred the mother or baby to specialists if they identified any health issues that required further attention.

### Tool development

We conducted a detailed desk review to develop a structured exit interview tool for maternal care service recipients and service providers. The desk review covered national and international guidelines, standard operating procedures, published journal articles, policy papers, reports and other grey literature. We systematically analysed findings from the desk review to identify key themes and gaps in maternal care services to structure the questionnaire into relevant sections, such as sociodemographic information, respectful maternity care, ease of getting services, satisfaction of mothers regarding ANC, delivery, and PNC services, and providers’ service delivery satisfaction.

### Questionnaire for maternal care service recipients

In the maternal care service recipient’s questionnaire, we had a total of 74 questions divided into eight sections including sociodemographic information (20 questions), respectful maternity care (eight questions), ease of getting service (two questions), ANC service (eight questions), during delivery service satisfaction (18 questions), PNC service satisfaction (eight questions), service-related satisfaction during discharge (two questions), and overall provider’s service satisfaction (eight questions). In all sections, excluding the sociodemographic information section, we measured pregnant women’s and recently delivered mothers’ satisfaction with maternal care services using a five-point Likert-type scale (where one referred to ‘very dissatisfied’ and five referred to ‘very satisfied’).

### Questionnaire for the maternal care service provider

In the maternal care service providers questionnaire, we had a total of 39 questions divided into five sections, including sociodemographic information (10 questions), working environment (18 questions), training (six questions), logistics support (three questions), and overall service satisfaction (two questions). In all the sections, excluding the sociodemographic information section, providers’ satisfaction level was measured using a five-point Likert-type scale. This scale ranged from one to five, where one indicated ‘very dissatisfied’ and five indicated ‘very satisfied’. This approach allowed us to quantify the providers’ satisfaction and identify specific areas where improvements are needed.

### Validation and finalisation

We piloted both tools in Netrokona DH for the demonstration of their effectiveness and usability. During the pilot phase, we interviewed 10 service recipients (five pregnant women and five recently delivered mothers) to seek experiential feedback. We interviewed four additional providers (two nurses and two midwives) to identify potential challenges associated with using the two tools. We continued with a meticulous compilation of the data derived from the tasking mandate of finding key insights for needed improvements. It was through this analysis that we examined the true implications of the tools, making minor changes to make them more user-friendly. After incorporating the feedback and making the required adjustments, we finalised the tools. This finalisation process involved thorough testing to confirm that the tools were ready for broader implementation.

### Data collection procedures

One male and one female doctor underwent a three-day comprehensive training to ensure proficiency in data collection. The principal investigator conducted training on data collection procedures, covering the following topics: the data collection instrument, the study’s objectives, and methods for conducting in-person interviews. The data collectors did not provide medical treatment to the women who received services in the facility. Before data collection, informed consent forms were obtained from both service recipients and providers. We collected data with proper privacy, and only the interviewer and participants were allowed to stay during the data collection period. Lastly, data was taken from the women in person during exit after they received one of the maternal care services. Participants were selected based on predefined inclusion and exclusion criteria.

This data was then entered into the International Centre for Diarrhoeal Disease Research, Bangladesh (icddr,b) in-house data management system. After completing the data entry, the research team (project physician and statistician) conducted a rigorous review and data quality check.

### Outcome variables

This study’s primary outcome variable was the percent mean (x̄) sum score of maternal and provider satisfaction with ANC, delivery, and PNC services provided by two DHs. To calculate the percent x̄ sum score of each component under each service, we utilised the following formula:

x̄ (%) sum score = (obtained score under that component × 100)/total score under that component

where the total score under that component equals the number of questions in that component ×5.

To calculate the overall percent, x̄ sum score under each service we utilised the following formula:

Overall percent x̄ sum score = (obtained score under that service × 100)/total score under that service

where the total score under that component equals the number of questions in that service ×5.

Additionally, the secondary outcome was the percentage of fully satisfied mothers in both midwifery care and without midwifery care (regular care) under each component, where the fully satisfied is defined as if a mother replied satisfied or very satisfied to a question.

### Data analysis plan

We conducted a descriptive analysis using STATA, version 15 (StataCorp LLC, College Station, Texas, USA). We investigated the background characteristics of pregnant women and mothers who went for maternal care services (ANC, delivery, PNC) in the facilities. To explore the background characteristics, we considered the following independent variables: age (<20 years, 20–30, and >30 years), educational qualification (up to primary, secondary, higher), and family income (<20 000 and ≥20 000). Moreover, we considered the obstetrics and gynaecological characteristics, such as gravida (primigravida, multigravida), para (nulliparous, multiparous), and gestational week (<37 weeks and ≥37 weeks). To explore the background characteristics of the providers we explored age (<30 years, 30–45, and >45), marital status (yes or no), educational level (degree, diploma, or other), duration of service (<5 years, 5–15, and >15). We compared the satisfaction level of the care receivers in midwifery care and regular care by using the percent x̄ sum score of each component of each. For the comparison, we used a forest plot with a 95% confidence interval (CI) to explore the difference between percent x̄ satisfaction score between midwifery care and regular care. Moreover, to see the significant difference between percent x̄ satisfaction score, we used an independent sample *t* test. Finally, we used linear regression to explore the impact of different characteristics on percent x̄ satisfaction score of the care receivers. We presented the results of linear regression as the x̄ difference between the satisfaction score and its 95% CI. All the analyses were one-tailed, and the *P*-value was set at 0.05.

### Ethical approval

We obtained ethical approval from the Institutional Review Board of icddr,b (P-23075). We also obtained permission from the Maternal, Newborn, Child, and Adolescent Health programme of the Directorate General of Health Services, Ministry of Health and Family Welfare, Bangladesh.

## RESULTS

**Figure 1:**
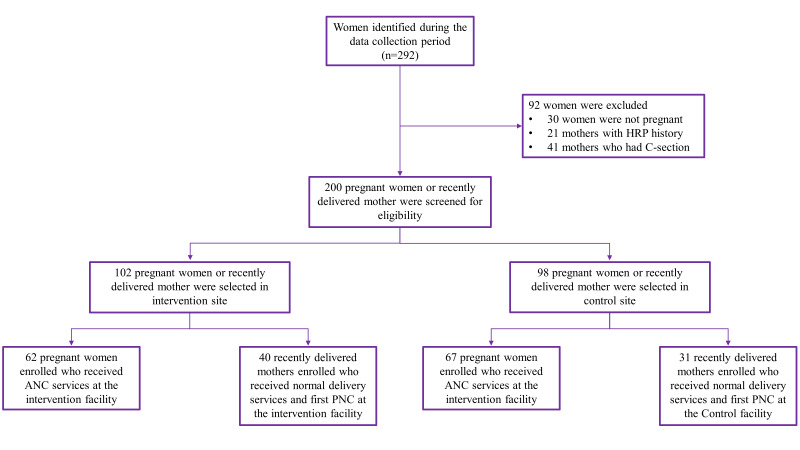
Participant enrolment flowchart.

We pre-screened a total of 292 women for participation in the study, and a total of 200 pregnant women or recently delivered mothers who visited the facility for ANC, delivery, or PNC services were screened for eligibility between 1 November 2023 and 15 December 2023 (**Figure 1**). Among the care receivers, 102 (51.0%) received midwifery care, and 98 (49.0%) received regular care. Of those who received midwifery care, 62 (60.8%) sought ANC services, and 40 (39.2%) sought delivery and PNC services. Instead, 67 (68.4%) of pregnant women who received regular care sought ANC service, and 31 (31.6%) recently delivered mothers received delivery and PNC service (**Table 1**).

**Table 1:** Background and obstetrics and gynaecological characteristics of pregnant women or recently delivered mothers in midwifery care and regular care*

	Midwifery care	Regular care	*P*-value
**ANC service**			
Age in years			0.500
*<20*	13 (21.0)	9 (13.4)	
*20–30*	43 (69.4)	52 (77.6)	
*>30*	6 (9.7)	6 (9.0)	
*Missing*	0 (0.0)	0 (0.0)	
Education			0.323
*No education*	2 (3.2)	1 (1.5)	
*Primary*	15 (24.2)	10 (15.0)	
*Secondary*	37 (59.7)	41 (61.2)	
*Higher*	8 (12.9)	15 (22.4)	
*Missing*	0 (0.0)	0 (0.0)	
Family income in BDT per month			0.141
*<20000*	42 (67.7)	36 (53.7)	
*≥20000*	17 (27.4)	22 (32.8)	
*Missing*	3 (4.8)	9 (13.4)	
Para			0.605
*Nulliparus*	24 (38.7)		
*Multiparus*	38 (61.3)		
Gravida			0.768
*Primigravida*	20 (32.3)	20 (29.9)	
*Multigravida*	42 (67.7)	47 (70.2)	
Gestational week			0.627
*<37*	49 (79.0)	52 (77.6)	
*≥37*	13 (21.0)	14 (20.9)	
*Missing*	0 (0.0)	1 (1.5)	
Total	62 (48.1)	67 (51.9)	
**Delivery service**			
Age in years			0.673
*<20*	8 (20.0)	7 (22.6)	
*20–30*	30 (75.0)	21 (67.7)	
*>30*	2 (5.0)	2 (6.5)	
*Missing*	0 (0.0)	1 (3.2)	
*Education*			0.125
*No education*	0 (0.0)	3 (9.7)	
*Primary*	13 (32.5)	5 (16.1)	
*Secondary*	18 (45.0)	18 (58.0)	
*Higher*	8 (20.0)	5 (16.1)	
*Missing*	1 (2.5)	0 (0)	
Family income in BDT per month			0.007
*<20000*	30 (75.0)	15 (48.4)	
*≥20000*	10 (25.0)	10 (32.2)	
*Missing*	0 (0)	6 (19.4)	
Para			0.377
*Nulliparus*	21 (52.5)	13 (41.9)	
*Multiparus*	19 (47.5)	18 (58.1)	
Gravida			0.377
*Primigravida*	21 (52.5)	13 (41.9)	
*Multigravida*	19 (47.5)	18 (58.1)	
Gestational week			0.491
*Preterm*	2 (5.0)	1 (3.2)	
*Term*	38 (95.0)	29 (93.6)	
*Missing*	0 (0.0)	1 (3.2)	
Total	40 (56.3)	31 (43.7)	
**PNC service**			
Age in years			0.673
*<20*	8 (20.0)	7 (22.6)	
*20–30*	30 (75.0)	21 (67.7)	
*>30*	2 (5.0)	2 (6.5)	
*Missing*	0 (0.0)	1 (3.2)	
Education			0.125
*No education*	0 (0.0)	3 (9.7)	
*Primary*	13 (32.5)	5 (16.1)	
*Secondary*	18 (45.0)	18 (58.1)	
*Higher*	8 (20.0)	5 (16.1)	
*Missing*	1 (2.5)	0 (0)	
Family income in BDT per month			0.007
*<20000*	30 (75.0)	15 (48.4)	
*≥20000*	10 (25.0)	10 (32.3)	
*Missing*	0 (0.0)	6 (19.4)	
Para			0.377
*Nulliparus*	21 (52.5)	13 (41.9)	
*Multiparus*	19 (47.5)	18 (58.1)	
Gravida			0.377
*Primigravida*	21 (52.5)	13 (41.9)	
*Multigravida*	19 (47.5)	18 (58.1)	
Gestational week			0.491
*Preterm*	2 (5.0)	1 (3.2)	
*Term*	38 (95.0)	29 (93.6)	
*Missing*	0 (0.0)	1 (3.2)	
Total	40 (56.3)	31 (43.7)	

ANC – antenatal care, PNC – postnatal

*Presented as n (%).

We assessed provider satisfaction by selecting midwives from the intervention facility and nurses from the control facility, both of whom were responsible for delivering ANC, delivery, and PNC services (**Table 2**).

**Table 2:** Background characteristics of providers in midwifery care and regular care*

Variables	Midwifery care	Regular care
Age in years		
*<30*	6 (100.0)	0 (0.0)
*30–45*	0 (0.0)	5 (62.5)
*>45*	0 (0.0)	3 (37.5)
Marital status		
*Yes*	5 (83.3)	8 (100.0)
*No*	1 (16.7)	0 (0.0)
Educational level		
*Degree*	0 (0.0)	1 (12.5)
*Diploma*	6 (100.00)	5 (62.5)
*Other courses*	0 (0.0)	2 (25.0)
Duration of services in the present workplace in years		
*<1*	3 (50.0)	4 (50.0)
*1–2*	2 (33.3)	4 (50.0)
*>2*	1 (16.7)	0 (0.0)
Total	6 (42.9)	8 (57.1)

*Presented as n (%).

### Patient satisfaction: pregnant women’s satisfaction with ANC services

**Figure 2:**
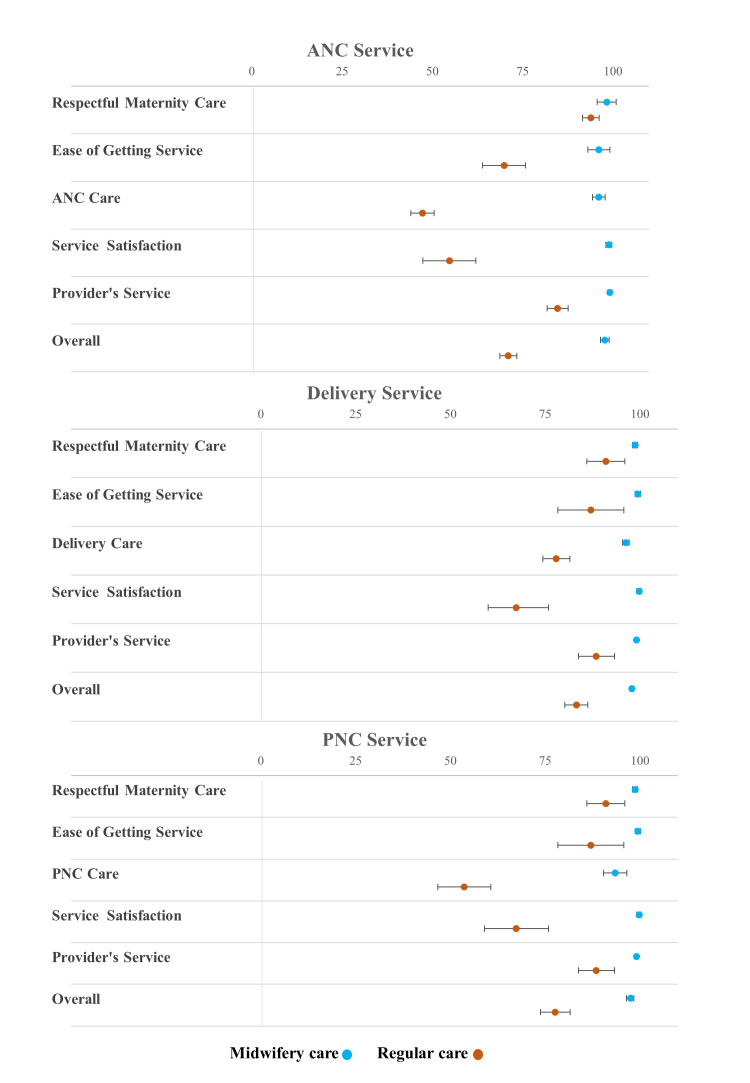
Mean satisfaction score of all components in ANC, delivery, and PNC service in midwifery care and regular care.

The overall mean satisfaction score of pregnant women who received service from midwifery care was significantly higher (x̄ = 97.8; 95% CI = 96.6–99.0) compared to pregnant women who received service from regular care (x̄ = 71.0; 95% CI = 68.7–73.4). In all five components of the ANC service, women who received ANC care from midwifery services had significantly higher satisfaction scores than women who received care from regular services (**Figure 2**). The satisfaction score of midwifery care for respectful maternity care was x̄ = 96.1 (95% CI = 93.1–99.2), for ease of getting service x̄ = 96.1 (95% CI = 94.4–97.9), for ANC care x̄ = 98.9 (95% CI = 98.1–99.7), and for service satisfaction during discharge and provider’s service x̄ = 99.2 (95% CI = 98.6, 99.7). On the other hand, in regular care satisfaction scores for the same components were x̄ = 93.9 (95% CI = 91.6–96.2), x̄ = 69.9 (95% CI = 63.9–75.8), x̄ = 47.3 (95% CI = 44.1–50.5), and x̄ = 54.8 (95% CI = 47.4, 62.1), respectively. In addition, the adjusted x̄ difference of the satisfaction score for each component in midwives providing care was significant compared to regular care, after adjusting for age, educational level, parity, and gestational week. Pregnant women in midwifery care were more likely to rate their overall satisfaction higher for the entire antenatal service (adjusted x̄ difference = 27.1; 95% CI = 24.4–29.7) compared to regular care. The adjusted differences in satisfaction scores between midwifery care and regular care were as follows: respectful maternity care x̄ = 4.2 (95% CI = 0.8–7.7), ease of getting service x̄ = 26.9 (95% CI = 19.9–33.9), ANC x̄ = 49.0 (95% CI = 45.3–52.8), service satisfaction during discharge x̄ = 44.2 (95% CI = 36.4–52.1), and provider’s service x̄ = 15.1 (95% CI = 12.0–18.2) (**Table 3**).

**Table 3:** Adjusted mean satisfaction score difference of all components in ANC, delivery, and PNC service between midwifery care and regular care*

Components	x̄ (95% CI)	*P*-value
ANC service		
*Respectful maternity care*	4.2 (0.8–7.7)	0.017
*Ease of getting service*	26.9 (19.9–33.9)	<0.001
*ANC care*	49.0 (45.3–52.8)	<0.001
*Service*	44.2 (36.4–52.1)	<0.001
*Provider’s service*	15.1 (12.0–18.2)	<0.001
*Overall*	27.1 (24.4–29.7)	<0.001
Delivery service		
*Respectful maternity care*	8.5 (3.7–13.3)	0.001
*Ease of getting service*	14.5 (6.4–22.5)	0.001
*Delivery care*	18.0 (15.0–21.0)	<0.001
*Service*	30.1 (22.5–37.7)	<0.001
*Provider’s service*	10.9 (6.4–15.4)	<0.001
*Overall*	14.7 (12.1–17.3)	<0.001
PNC service		
*Respectful maternity care*	8.5 (3.7–13.3)	0.001
*Ease of getting service*	14.5 (6.4–22.5)	0.001
*PNC care*	38.6 (31.2–46.0)	<0.001
*Service*	30.1 (22.5–37.7)	<0.001
*Provider’s service*	10.9 (6.4–15.4)	<0.001
*Overall*	19.8 (16.1–23.4)	<0.001

ANC – antenatal care, CI – confidence interval, PNC – postnatal care, x̄ – mean

### Patient satisfaction: recently delivered mothers’ satisfaction with the delivery service

Mothers who received service from midwifery care had a significantly higher score (x̄ = 97.9; 95% CI = 97.4–98.3) compared to those who received service from regular care (x̄ = 83.3; 95% CI = 80.2–86.3). Among all the components, the service satisfaction score for midwifery care was significantly higher than for regular care (**Figure 2**). Additionally, the delivery care component from midwifery care had the second-highest mean satisfaction score, and significantly higher than regular care. The satisfaction score for respectful maternity care was x̄ = 98.8 (95% CI = 98.1–99.4) under midwifery care, compared to x̄ = 91.0 (95% CI = 86.0–96.0) under regular care. For ease of getting service, the score was x̄ = 99.5 (95% CI = 98.8–100.2) with midwifery care and x̄ = 87.1 (95% CI = 78.4–95.8) with regular care. In delivery care, midwifery care scored x̄ = 96.3 (95% CI = 95.4–97.2), whereas regular care scored x̄ = 77.9 (95% CI = 74.4–81.5). Satisfaction during discharge was x̄ = 99.8 (95% CI = 99.2–100.3) for midwifery care, compared to x̄ = 67.4 (95% CI = 60.0–75.9) for regular care. Lastly, for providers’ service, midwifery care scored x̄ = 99.1 (95% CI = 98.7–99.5) *vs.* x̄ = 88.5 (95% CI = 83.8–93.3) in regular care. In addition, the adjusted x̄ difference of the satisfaction score for each component in midwives providing care was significant compared to regular care, after adjusting for age, educational level, parity, and gestational week. Mothers in midwifery care were more likely to rate their overall satisfaction higher for overall delivery service (adjusted mean difference = 14.7; 95% CI = 12.1–17.3) compared to regular care. The adjusted differences in satisfaction scores between midwifery care and regular care were x̄ = 8.5 (95% CI = 3.7–13.3) for respectful maternity care, x̄ = 14.5 (95% CI = 6.4–22.5) for ease of getting service, x̄ = 18.0 (95% CI = 15.0–21.0) for delivery care, x̄ = 30.1 (95% CI = 22.5–37.7) for service satisfaction during discharge, and x̄ = 10.9 (95% CI = 6.4–15.4) for provider’s service. (**Table 3**).

### Patient satisfaction: recently delivered mothers’ satisfaction with the PNC service

Mothers who received service from midwifery reported a significantly higher overall satisfaction score (x̄ = 97.5; 95% CI = 96.5–98.4) compared to those who received regular care (x̄ = 77.7; 95% CI = 73.8–81.6). In all five components, mothers who received service from midwifery care for PNC service had significantly higher x̄ satisfaction scores compared to mothers who went to regular care (**Figure 2**). The satisfaction score for respectful maternity care was x̄ = 98.8 (95% CI = 98.1–99.4) with midwifery care compared to x̄ = 91.0 (95% CI = 86.0–96.0) with regular care. For ease of getting service, the scores were x̄ = 99.5 (95% CI = 98.8–100.2) *vs.* x̄ = 87.1 (95% CI = 78.4–95.8), and for PNC care x̄ = 93.5 (95% CI = 90.4–96.6) *vs.* x̄ = 53.7 (95% CI = 46.7–60.7). Service satisfaction during discharge scored x̄ = 99.8 (95% CI = 99.2–100.3) with midwifery care compared to x̄ = 67.4 (95% CI = 60.0–75.9) with regular care, while provider’s service satisfaction was x̄ = 99.1 (95% CI = 98.7–99.5) *vs.* x̄ = 88.5 (95% CI = 83.8–93.3).

In addition, the adjusted mean difference of the satisfaction score for each component in midwives providing care was significant compared to regular care, after adjusting for age, educational level, parity, and gestational week. Mothers in midwifery care were more likely to rate their overall satisfaction higher for the entire PNC service (adjusted mean difference = 19.8; 95% CI = 16.1–23.4) compared to regular care. The adjusted differences in satisfaction scores between midwifery care and regular care were x̄ = 8.5 (95% CI = 3.7–13.3) for respectful maternity care, x̄ = 14.5 (95% CI = 6.4–22.5) for ease of getting service, x̄ = 38.6 (95% CI = 31.2–46.0) for PNC care, x̄ = 30.1 (95% CI = 22.5–37.7) for service satisfaction during discharge, and x̄ = 10.9 (95% CI = 6.4–15.4) for provider’s service (**Table 3**).

### Provider’s satisfaction

**Figure 3:**
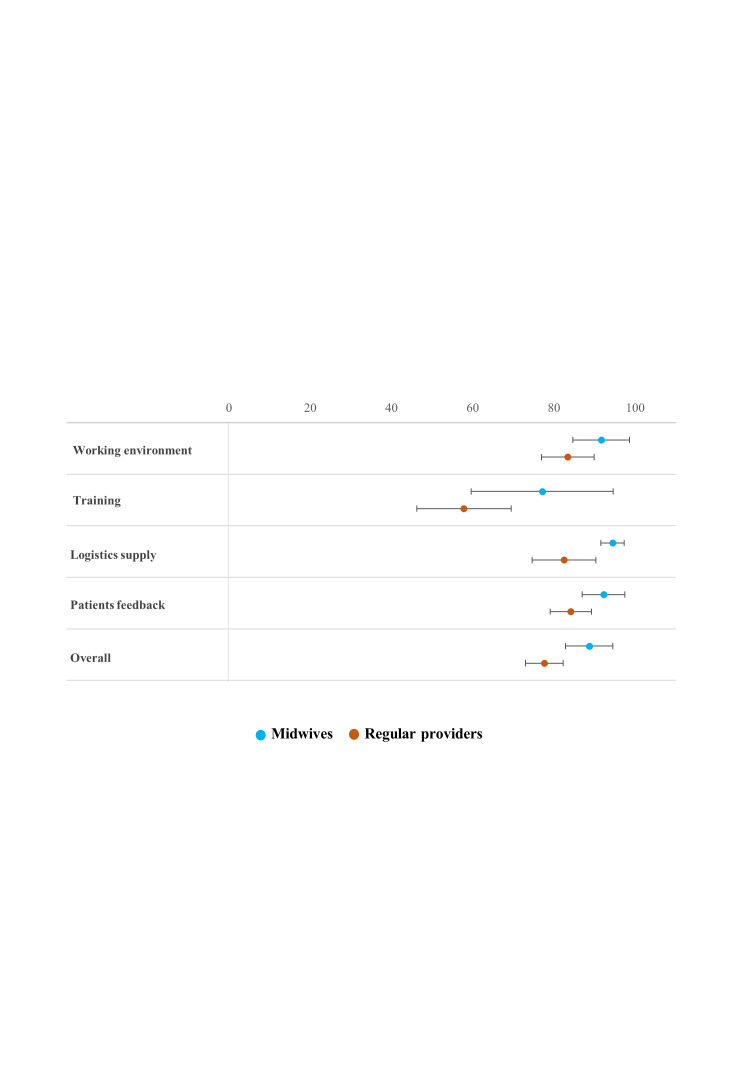
Mean satisfaction score of all components of midwives (in midwifery care) and providers (in regular care).

The overall satisfaction score of midwives (x̄ = 88.7; 95% CI = 82.9–94.5) was significantly higher compared to providers in regular care (x̄ = 77.7; 95% CI = 73.0–82.3). In all four components, midwives had a higher satisfaction score compared to providers in regular care (**Figure 3**). The satisfaction score for the working environment was x̄ = 91.7 (95% CI = 84.7–98.7) among midwives, compared to x̄ = 83.5 (95% CI = 77.0–90.0) among regular care providers. For training, the scores were x̄ = 77.1 (95% CI = 59.7–94.6) for midwives and x̄ = 57.9 (95% CI = 46.2–69.5) for regular providers. Regarding logistic supply, midwives reported a score of x̄ = 94.4 (95% CI = 91.6–97.3), while regular providers scored x̄ = 82.5 (95% CI = 74.7–90.3). For patients’ feedback, satisfaction was x̄ = 92.2 (95% CI = 87.0–97.5) among midwives and x̄ = 84.2 (95% CI = 79.1–89.3) among regular care providers.

## DISCUSSION

This is the first study in Bangladesh to examine both provider and recipient satisfaction with midwifery care. Our findings indicate that the introduction of midwifery care at secondary-level health care facilities significantly improves satisfaction levels among pregnant women and new mothers during ANC, PNC, and delivery services, as well as among health care providers who provide the services.

In particular, women who received ANC services from facilities with midwives reported significantly higher satisfaction than those in settings without midwifery support. This finding is consistent with research by Shields et al. in Australia, which also highlighted the association between midwifery care and higher satisfaction during antenatal visits [26]. Moreover, a study in Ireland found that midwifery care maintained comparable levels of ANC utilisation [27], further reinforcing that the involvement of midwives enhances patient satisfaction without diminishing access to care. The higher satisfaction observed among women receiving midwifery care can be attributed to several interconnected factors inherent in the midwifery model. Comprehensive counselling on physical and mental support, along with delivery planning, helps women feel better prepared and assured. Midwives provide personalised guidance on selecting the appropriate delivery place and health care provider, often using behaviour change communication materials to improve understanding. Their attentive listening and clear follow-up instructions foster a strong rapport, further enhanced by consistent advice on health improvement. The availability of midwives, longer caregiving time, and favourable midwife-to-patient ratios enable more focused and individualised care, contributing to higher patient satisfaction. In contrast, nurse-led care may involve more medical interventions, which, although necessary, may not always align with a mother’s preference for a natural birth experience. Privacy, comfort, and efficient labour management are crucial in both models, but midwifery care emphasises these aspects more. Additionally, factors like clear communication, effective pain management, and the presence of a companion during childbirth further enhance maternal satisfaction. This is consistent with findings from a study in Bangladesh, which highlighted that a significant portion of nurses' working hours in government hospital settings were classified as unproductive time, spent away from patient care areas, further limiting their ability to provide continuous and personalised care [28]. This unproductive time can limit the quality and continuity of care that nurses can provide, further emphasising the unique value of the midwifery model in enhancing patient satisfaction.

Midwifery care significantly enhanced maternal satisfaction during delivery. Women reported higher satisfaction with facilities that offered midwifery services compared to those served by nurses. This aligns with Australian studies showing greater satisfaction with midwifery care during hospital intrapartum care [29,30]. The satisfaction derived from midwifery care can be attributed to several factors. Midwives often explain the normal vaginal delivery procedure and treatment plan in detail, discuss various birth positions, and allow women to choose their preferred birthing method. They also support the presence of a labour companion, briefing them on their role and providing cognitive and emotional support throughout the process. Effective communication with the health care team and patient attendees, along with the use of non-pharmacological pain management techniques, further enhances the birthing experience. Additionally, midwives offer guidance on breastfeeding techniques within the first hour after birth and advise against bathing the newborn within the first 72 hours. These practices enhance the birthing experience and highlight the importance of integrating midwifery services into health care systems to improve patient experiences and outcomes during intrapartum care [31,32].

The x̄ satisfaction score of pregnant women who received PNC services from facilities offering midwifery care was significantly higher compared to those who received care from facilities without midwifery services. This aligns with findings from an Australian study, which reported higher overall satisfaction ratings for hospital postpartum care associated with midwifery care [29,30]. We further identified lower satisfaction levels with PNC services in regular care facilities, particularly in key areas such as explaining the importance of postnatal care, discussing post-delivery physical and mental changes, providing exclusive breastfeeding counselling, and offering guidance on maternal and newborn care. In contrast, midwifery care consistently performed better in these aspects, likely due to the core elements of the midwifery model. Midwives were more likely to provide detailed explanations about PNC, engage in discussions about postpartum physical and mental changes, and offer personalised exclusive breastfeeding counselling. They also utilised behaviour change communication materials to educate mothers on danger signs, while offering comprehensive briefings on maternal hygiene, nutrition, newborn care, and postpartum family planning. These personalised interactions, along with better communication, support, and continuity of care, contributed to the higher satisfaction observed among women receiving midwifery care. A systematic review indicated that midwifery care is associated with better maternal outcomes, including lower rates of postpartum haemorrhage and higher rates of successful vaginal births [12]. The ability of women to connect with midwives during the early postnatal period helped them overcome barriers and successfully transition to motherhood [33]. Midwives emphasised the pregnancy to postnatal continuity as crucial to providing care based on individual needs [34].

In our study, the impact of midwifery-led care extended beyond patient satisfaction, as midwives themselves reported significantly higher job satisfaction compared to nurses in regular care settings. Midwives in intervention facilities expressed greater satisfaction with their working environment, the availability of resources, necessary training, and patient feedback mechanisms, which echoes findings from the Netherlands, where midwives also reported high levels of job satisfaction, though the reasons differed across settings [35]. This comparison is crucial, as our study is one of the first to examine job satisfaction among midwives and nurses in secondary-level facilities that offer midwifery-led *vs.* regular care. The higher job satisfaction among midwives may stem from their specialised focus on maternal care, allowing them to concentrate more fully on pregnant women and new mothers. In contrast, nurses in general care often juggle multiple patient types, which can lead to lower job satisfaction and compromised care quality. The research underscores the importance of provider job satisfaction, highlighting that satisfied health care providers are more likely to deliver high-quality care, stay longer in their positions, and contribute to overall workplace morale and productivity [36]. Therefore, ensuring high levels of job satisfaction, particularly through specialised roles like midwifery, is essential for maintaining a stable, effective health care system, reducing burnout, and promoting better patient outcomes. Midwifery-led care is to be integrated into national-level policy planning, considering scaling up at the secondary and tertiary facilities in Bangladesh. A collaborative effort among global, regional, and national stakeholders is critical in order to achieve the synchronisation of research, evidence-informed policies and sharing knowledge with similar settings.

### Strengths and limitations

This study is pioneering in Bangladesh, being the first to examine both provider and recipient satisfaction with midwifery care in secondary-level health care facilities. It offers a comprehensive analysis by evaluating satisfaction levels across ANC, PNC, and delivery services, providing a holistic view of the impact of midwifery care. The comparative approach between midwives and nurses highlights the unique benefits of midwifery care, such as personalised and continuous support. Additionally, the study’s findings are consistent with international research, reinforcing the global relevance of midwifery care in improving maternal and neonatal outcomes.

The study’s geographical scope is limited to purposively selected secondary-level health care facilities in Bangladesh, which may introduce potential biases and may not fully represent the situation in primary or tertiary care settings or other countries. For future studies, data-driven approaches can be employed to identify and address such biases. The sample size and diversity may also be limited, potentially affecting the generalisability of the findings to all regions and populations within Bangladesh. Hence, due to the small sample size of providers, the impact of job roles, responsibilities, and workload on satisfaction could not be determined. There may be inherent biases in self-reported satisfaction levels from both providers and recipients, which could influence the study’s outcomes. Furthermore, the study may not account for all resource constraints and systemic challenges faced by health care facilities, which can impact the implementation and effectiveness of midwifery care.

## CONCLUSIONS

In conclusion, this pioneering study in Bangladesh demonstrates that the introduction of midwifery care at secondary-level health care facilities significantly enhances satisfaction among pregnant women, new mothers, and health care providers. These results underscore the importance of expanding midwifery services to higher-level health care facilities, to further improve maternal and neonatal health outcomes and overall patient satisfaction. Implementing these changes in government policy could lead to a more effective and compassionate health care system, benefiting both providers and recipients.

## Additional material


Online Supplementary Document
