# Supplementary material for: Effect of involvement of midwives in maternal care on patient and provider satisfaction in secondary-level public health facilities in Bangladesh: a comparative quasi-experimental study
Source: J Glob Health. 2025 Jul 4;15:04183. doi: 10.7189/jogh.15.04183 (PMC12231468; doi:10.7189/jogh.15.04183)

Supplement to: Mahmood HR, Hossain L, Sayeed A, Azrin F, Mallick T, Hayder T, Ahmed A, Jabeen S, Tonmon TT, Rahman MM, Hassan AKMM, Siddique MAB, Zaman S, Rahman A, Murshid HB, Nadia N, Mahmud M, Alim MA, Hoque DME, Hasan ASM, Arifeen SE, Rahman AE, Rasghuvanshi VS. Effect of involvement of midwives in maternal care on patient and provider satisfaction in secondary-level public health facilities in Bangladesh: a comparative quasi-experimental study. J Glob Health. 2025;15:04183.

Figure S1. Midwives' shift schedule and distribution in the intervention facility (Sunamganj DH).

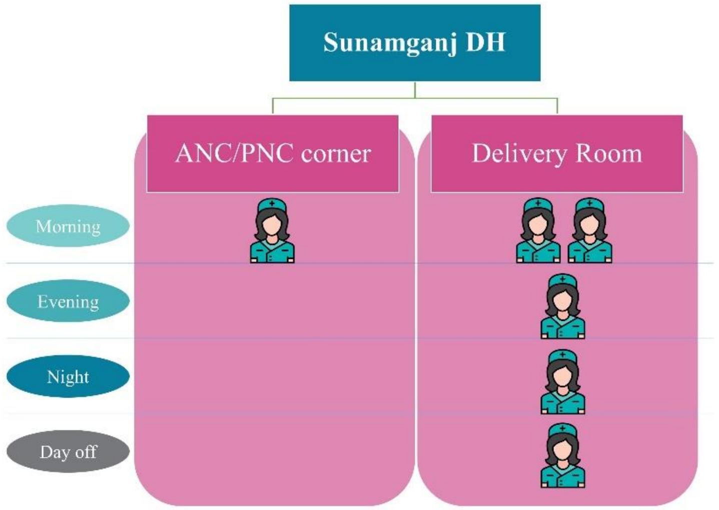

Figure S2. List of responsibilities of midwives.

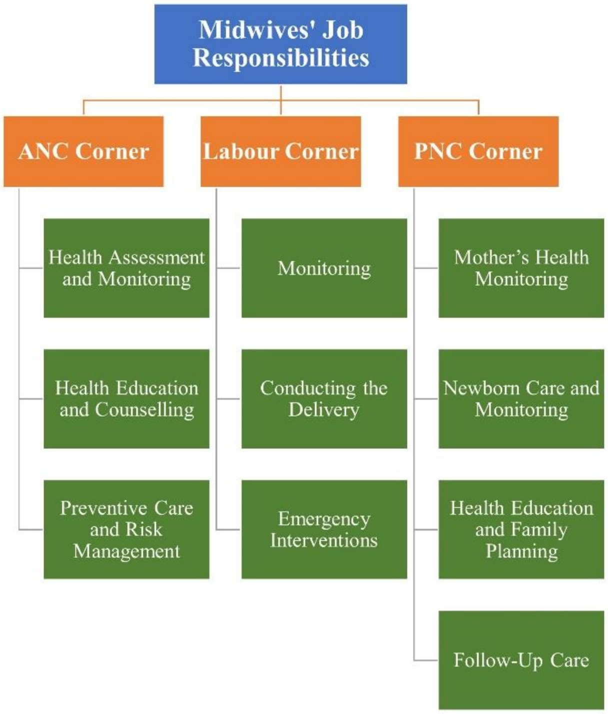

Supplement: Online Supplementary Document [file jogh-15-04183-s001.pdf]
